# Primary tubercular caecal perforation: a rare clinical entity

**DOI:** 10.1186/1471-2482-10-12

**Published:** 2010-03-31

**Authors:** Devendra K Jain, Gaurav Aggarwal, Parvinder S Lubana, Sonia Moses, Nitin Joshi

**Affiliations:** 1 Department of Surgery, M.G.M. Medical College and M.Y. Hospital, Indore, Madhya Pradesh -452001, India

## Abstract

**Background:**

Intestinal tuberculosis is a common problem in endemic areas, causing considerable morbidity and mortality. An isolated primary caecal perforation of tubercular origin is exceptionally uncommon.

**Case presentation:**

We report the case of a 39 year old male who presented with features of perforation peritonitis, which on laparotomy revealed a caecal perforation with a dusky appendix. A standard right hemicolectomy with ileostomy and peritoneal toileting was done. Histopathology revealed multiple transmural caseating granulomas with Langerhans-type giant cells and acid-fast bacilli, consistent with tuberculosis, present only in the caecum.

**Conclusions:**

We report this extremely rare presentation of primary caecal tuberculosis to sensitize the medical fraternity to its rare occurrence, which will be of paramount importance owing to the increasing incidence of tuberculosis all over the world, especially among the developing countries.

## Background

Intestinal tuberculosis is an extremely common occurrence in India. Any portion of the gastrointestinal tract may be affected by it, and various pathogenic mechanisms are involved. However, a primary isolated caecal perforation due to tuberculosis is an unusual occurrence, without the concomitant involvement of the ileum or ascending colon. We report this extremely rare presentation, the diagnosis of which was made retrospectively, with a view to sensitize the entire world to this entity.

## Case Presentation

**Figure 1:**
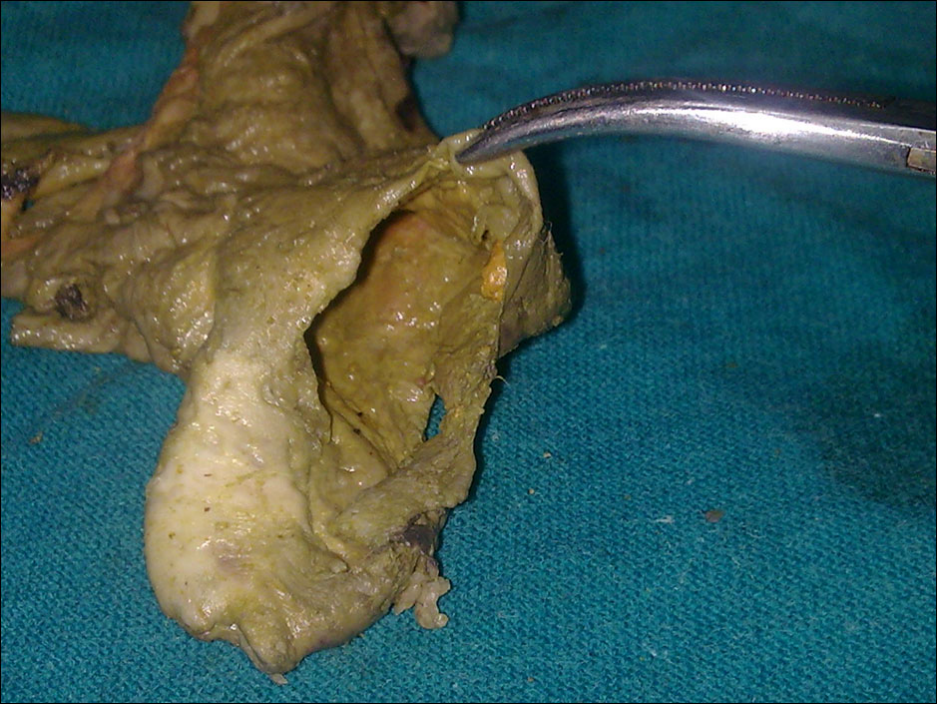
**Post operative specimen, clearly demonstrating the caecal perforation**.

**Figure 2:**
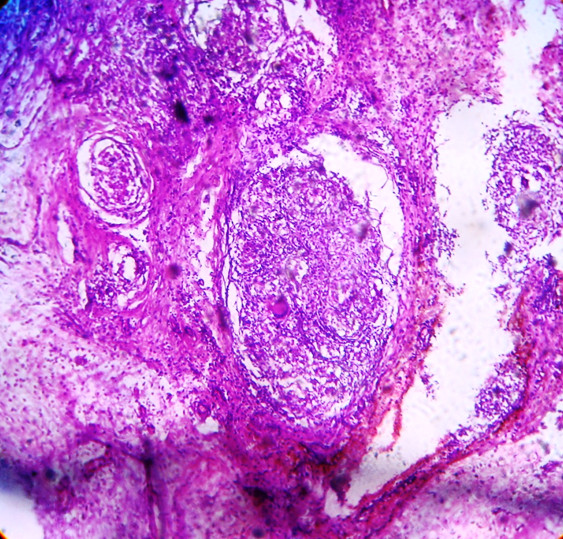
**Microscopic appearance, showing evidence of granulomatous tuberculous lesion**.

A 39 year old male presented to the emergency department, in the month of May 2009, with complaints of right lower quadrant abdominal pain and low grade fever of 2 days duration. He was a chronic alcoholic since 10 years and had no other significant prior medical, surgical history, or any evidence of a traumatic event. On examination, the patient had tachycardia, and slight tenderness and guarding, localized to the right iliac fossa. The rest of the abdomen was non tender. His routine blood tests were within normal limits, with completely normal counts. His HIV and HBsAg tests were negative. An x ray of his abdomen (erect film) showed a ground glass appearance. Ultrasound examination of his abdomen however revealed features suggestive of terminal ileitis or ruptured appendix (caecal mural thickening, with minimum peri-appendicular fluid) with a normal liver. Clinical suspicion was of a recently ruptured appendix. The patient was taken up for an emergency laparotomy, via a right paramedian incision (considering his USG findings). Intra operatively, a 3 × 2 cm caecal perforation was present along the antero lateral wall with a dusky appendix attached to the caecum via a narrow base. There was no peritoneal contamination and the rest of the bowel appeared completely normal. The liver and spleen were normal and there was no lymphadenopathy. A right hemi colectomy with ileostomy and transverse colonic mucous fistula was done and the specimen was sent for a histopathological diagnosis(figure 1). A peritoneal biopsy was also concomitantly taken. The histopathology reported features of tubercular granulomatous lesion with secondary acute infection with surgical margins showing non-specific inflammation, presence of caseation with langerhans giant cells(figure 2) as well as acid fast bacilli-consistent with tubercular enteritis, in the caecum, with a completely normal ileum and ascending colon. Moreover, the peritoneal biopsy was found to be non specific, with no features of tuberculosis.

We then performed a retrospective analysis of our management effort towards the patient, the x ray chest was normal, mantoux test was negative, ESR was 18 mm at 1 hour, and sputum was negative(three samples). The patient was started on anti-tubercular therapy (DOTS Category 1 - incorporating 4 drugs, viz. isoniazide, rifampicin, pyrizinamide and ethambutol for initial 2 months of the intensive phase, and isoniazide and rifampicin for the next 4 months of the continuation phase), with satisfactory results as regards the improvement in the overall patient condition, with 6 kgs weight gain over two months. The patient became comfortable, ambulatory; Ileostomy was functional and viable and main wound healing occurred via secondary intention. The patient was followed up on a regular 2 weekly basis with ileostomy closure performed after 3 months, without incident.

## Discussion

Isolated caecal perforation, that too tubercular in origin, is a rare entity. Caecal perforations are commonly encountered as a part and parcel of various associated disease processes, in accordance with Laplace's law. Laplace's law dictates that the intraluminal pressure needed to stretch the wall of a hollow tube is inversely proportional to its radius. The cecum has the largest diameter of the colon, and as such, requires the least amount of pressure to distend [1-3]. The diameter of the cecum in which perforation is imminent has been estimated to be between 9 cm and 16 cm [4].

Caecal perforations are usually seen associated with entities such as diverticular disease, inflammatory bowel diseases, ogilville syndrome[2], closed loop obstructions[5], pancreatic carcinomas[6], colorectal cancers[7], hirschsprung's disease[8], etc, in the presence of a patent ileo-caecal valve.

In dealing with an emergency colonic obstruction leading to a caecal perforation, few authors favour some form of primary anastomosis as opposed to others favoring creating of an initial stoma after a standard right hemi-colectomy. Many others have recommended subtotal or extended right hemicolectomy for emergency left colon or distal transverse colon obstruction. This is advisable in cecal perforations complicating large bowel obstruction, and in those patients who have a high risk of a metachronous lesion developing[9]. This is also advisable if the proximal colon cannot be evaluated before operation by colonoscopy or barium enema[10]. Though tuberculosis is eminently treatable medically, surgery is still often required for the suspected or confirmed abdominal variety, presenting with complications or as any atypical diagnostic problems [11].

Ileocecal tuberculosis has always been elusive to physicians, patients often ending in the surgical ward with acute or subacute obstruction, intractable vomiting and diarrhea and often chronic abdominal masses. The precise incidence of gastrointestinal tuberculosis is not known, due to a lack of random samples of populations studied. However, the reported incidence varies from 0.02% to 5.1% in various autopsy series[12]. Furthermore, the exact mechanism responsible for an isolated involvement of the caecum remains unknown. Clinicians require an extremely high index of suspicion to be able to diagnose these cases at an early stage, based on clinical signs and symptoms. The rarity of this case, as encountered by us, lies in the occurrence of an isolated primary caecal tubercular perforation, without any prior evidence, investigative or clinical finding of the same.

## Conclusion

A spontaneous primary caecal perforation can be tubercular in origin even in the absence of features suggestive of the same, and thus this unusual entity must be borne in mind for '*what the mind knows is what the eyes see'*.

## Consent

The patient has granted his written and informed consent for publication of this work.

## Competing interests

The authors declare that they have no competing interests.

## Authors' contributions

PSL, SM, GA, NJ were the operating team of surgeons and DKJ was the overall team incharge, under whose guidance the entire surgery was performed and who guided the subsequent management of the patient. All authors have read and approved the manuscript.

## Pre-publication history

The pre-publication history for this paper can be accessed here:

http://www.biomedcentral.com/1471-2482/10/12/prepub
